# Oxidative Stress Protection and Anti-Inflammatory Activity of Polyphenolic Fraction from *Urtica dioica*: In Vitro Study Using Human Skin Cells

**DOI:** 10.3390/molecules30122515

**Published:** 2025-06-09

**Authors:** Katarzyna Wójcik-Borowska, Weronika Wójciak, Magdalena Żuk, Piotr Luchowski, Agnieszka Skalska-Kamińska, Wiktoria Pacuła, Ireneusz Sowa, Magdalena Wójciak

**Affiliations:** 1 Department of Child Neurology, Medical University of Lublin, 20-093 Lublin, Poland; kwojcikborowska@gmail.com; 2 Department of Analytical Chemistry, Medical University of Lublin, Chodźki 4a, 20-093 Lublin, Poland; weronikawojciak01@gmail.com (W.W.); magdalena.zu25@gmail.com (M.Ż.); agnieszka.skalska-kaminska@umlub.pl (A.S.-K.); wiktoria.pacula@umlub.pl (W.P.); i.sowa@umlub.pl (I.S.); 3 Department of Neurology and Neurological Nursing, Medical University of Lublin, 20-954 Lublin, Poland; piotr.luchowski@umlub.pl

**Keywords:** nettle, polyphenols, anti-inflammatory, oxidative stress, antioxidant enzymes

## Abstract

Polyphenols are valuable contributors to skin health, offering potent antioxidant and anti-inflammatory effects that help counteract the process of inflammaging. According to the literature, *Urtica dioica* L. is a rich source of polyphenolic compounds, suggesting its potential for applications in cosmetology and dermatology. This study aimed to evaluate the antioxidant and anti-inflammatory activity of polyphenol-rich fractions isolated from *U. dioica* leaves (UdLs) and flowers (UdFs) using human skin cells subjected to oxidative stress and lipopolysaccharide (LPS) stimulation, respectively. Extracts were obtained via an accelerated solvent extraction and further purified by a solid-phase extraction to concentrate their polyphenolic content. Their chemical composition was analyzed using UPLC-DAD-MS. Biological activity was assessed through cytotoxicity assays (NR and MTT), chemical and cellular antioxidant assays (DPPH, ABTS, FRAP, CUPRAC, TPC, and H₂DCFDA), an evaluation of antioxidant enzyme activity (SOD, CAT), lipid peroxidation (MDA), and cytokine production (IL-1β, IL-6, IL-10). Our study showed that both fractions were abundant in phenolic compounds, with chlorogenic acid identified as the predominant constituent. UdLs contained higher levels of phenolic acids, whereas the UdF was richer in flavonoids, particularly derivatives of isorhamnetin. Both the UdL and UdF were non-cytotoxic and exhibited strong radical scavenging potential, with the UdL being slightly more effective. They significantly reduced intracellular ROS levels, enhanced the activity of antioxidant enzymes, and attenuated lipid peroxidation in cells exposed to oxidative stress. Moreover, both fractions reduced the secretion of pro-inflammatory cytokines in LPS and H_2_O_2_-stimulated fibroblasts. These results highlight the potential of polyphenolic fractions derived from *U. dioica* leaves and flowers as multifunctional ingredients for anti-aging and skin-protective cosmetics.

## 1. Introduction

Polyphenols play a significant role in promoting skin health and counteracting the visible and biological effects of aging. Their wide-ranging benefits include powerful antioxidant and anti-inflammatory activities, which make them highly desirable in cosmetic formulations. By neutralizing reactive oxygen species (ROS), they help prevent oxidative stress, while their ability to alleviate inflammatory states allows them to slow down the negative effects associated with inflammaging [1]. Structurally related compounds, such as coumarins, isocoumarins, and their glycosylated analogs, also exhibit significant antioxidant and anti-inflammatory activity, highlighting the importance of this class of metabolites in aging-related applications [2,3].

Inflammaging refers to the chronic process associated with aging, which contributes to skin deterioration and the progression of age-related conditions, including wrinkles, a loss of elasticity, hyperpigmentation, and an increased susceptibility to infections. External environmental factors, such as pathogens, ultraviolet (UV) radiation, and air pollution, can accelerate inflammaging because they promote ROS production and trigger oxidative damage in cells [4]. Taking the above into account, plants with a high polyphenol content are considered promising cosmetic additives. *Urtica dioica* L. (Urticaceae), commonly referred to as stinging nettle, is an example of such a plant. It ranks among the most extensively used medicinal plants, valued for its wide range of health-promoting properties, non-toxic nature, and easy availability [5,6]. The plant is a rich source of various phytochemicals from the class of secondary metabolites, including terpenes, phenolic acids, flavonoids, chlorophylls, carotenoids, and fatty acids [7,8,9,10,11].

*Urtica dioica* is a species with a well-established significance in folk medicine in many countries. Aqueous and alcoholic preparations from nettle have been used for the treatment of anemia; nasal and menstrual hemorrhages; rheumatism; urinary tract disorders, including gout, bladder, and kidney problems; and to enhance lactation. Externally, nettles show beneficial effects on skin and support the treatment of skin eczema and alleviate inflammation [12,13,14]. Contemporary medicine also values its potential. Researchers have found out that stinging nettle shows anti-inflammatory, anti-proliferative, antioxidant, antibacterial, hypolipemic, and analgesic activity [15,16,17,18]. Furthermore, there are some in vitro investigations indicating the anticancer properties of *U. dioica* extracts [19,20,21]. Research has shown that it possesses significant wound-healing properties that could accelerate the repair of damaged skin [22].

Our previous study revealed that *U. dioica* extract possesses significant ROS scavenging activity and protects human skin fibroblasts from the cytotoxicity of free radicals [23]. It can be supposed that this effect is attributed to polyphenolic compounds, which are known for their strong antioxidant activity [24,25]. Thus, this work expands on our investigation into the effects of nettle on skin cells, focusing on the polyphenol-rich fraction isolated from nettle leaves and flowers.

The aim of this work was to evaluate the antioxidant and anti-inflammatory potential of the polyphenol fractions using human keratinocytes and fibroblasts exposed to oxidative stress and lipopolysaccharide as a model. The fraction was qualitatively and quantitatively characterized using ultra-high-performance liquid chromatography with a photodiode array detection and mass spectrometry (UPLC-DAD-MS).

## 2. Results

### 2.1. Chemical Composition of Fraction Isolated from Urtica dioica Flower and Leaves

The accelerated solvent extraction (ASE) technique was used to prepare the extracts from *Urtica dioica*, which were further subjected to the solid-phase extraction in order to remove sugars and lipophilic compounds and concentrate polyphenolic components. ASE conditions were based on the paper of Repajić et al. [26].

**Figure 1:**
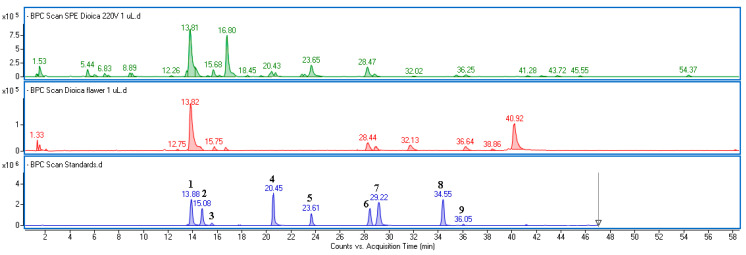
The base peak chromatogram obtained in the negative ionization mode of the polyphenolic fraction isolated from the ethanol–water extract of *U. dioica* leaves (green line), flowers (red line), and a mixture of standards (blue line), including 1—5-*O*-caffeoylquinic acid, 2—caffeic acid, 3—4-*O*-caffeoylquinic acid, 4—*p*-coumaric acid, 5—ferulic acid, 6—quercetin-3-*O*-rutinoside, 7—quercetin 3-*O*-glucoside, 8—kaempferol 3-*O*-rutinoside, and 9—isorhamnetin 3-*O*-rutinoside.

The obtained fractions from leaves (UdLs) and flowers (UdFs) were qualitatively and quantitatively characterized using UPLC-DAD-MS. The identity of the compounds was established based on MS and UV-Vis spectra extracted from individual peaks recorded on the base peak chromatogram (BPC) and DAD chromatograms (Figure 1 and Figure S1). Chemical formulas were determined using MassHunter software (version 10.0). A comparison of spectral data with those from commercially available standards or from the literature data was used to confirm identifications [26,27,28].

The chemical profiles of the fractions differed significantly. The UdL contained a higher amount of phenolic acids, whereas the UdF was richer in flavonoid compounds. In both extracts, the main peak was identified as an ester of caffeic and quinic acid (5-*O*-caffeoylquinic acid), based on its characteristic UV-Vis spectrum and fragment ions with *m*/*z* = 179 (corresponding to caffeic acid) and *m*/*z*–H = 191 (corresponding to quinic acid). The UdL also exhibited a high concentration of caffeoylmalic acid (*m*/*z*–H = 295), caffeic acid (*m*/*z*–H = 179), and p-coumaric acid (*m*/*z*–H = 163). These phenolic acids accounted for over 81% of the polyphenolic components found in UdLs. Flavonoids in the UdF were represented mainly by isorhamnetin, quercetin, and kaempferol derivatives, including rutinosides and glucosides. In addition to chlorogenic acid, the fraction isolated from the flower extract contained a significant amount of acetylated glucoside derivatives with isorhamnetin acetylglucoside being the predominant flavonoid. These compounds were present only in trace amounts in UdLs. Examples of the MS and UV-Vis spectra of the main identified components are provided in the Supplementary Material (Figure S2). The MS data of the predominant constituents, along with the quantification results expressed as mg per gram of dried extract, are summarized in Table 1.

### 2.2. Cytotoxicity Test

In the first part of this study, human skin fibroblasts and keratinocytes were incubated with different concentrations of UdL and UdF extracts to assess their impact on the cell viability and proliferation using two complementary assays: the neutral red (NR) and MTT assays. The NR assay evaluates the ability of living cells to uptake and accumulate the dye in lysosomes, while the MTT assay measures the metabolic activity, as NAD(P)H-dependent cellular oxidoreductases in viable cells are capable of converting yellow tetrazolium salt into purple formazan.

**Figure 2:**
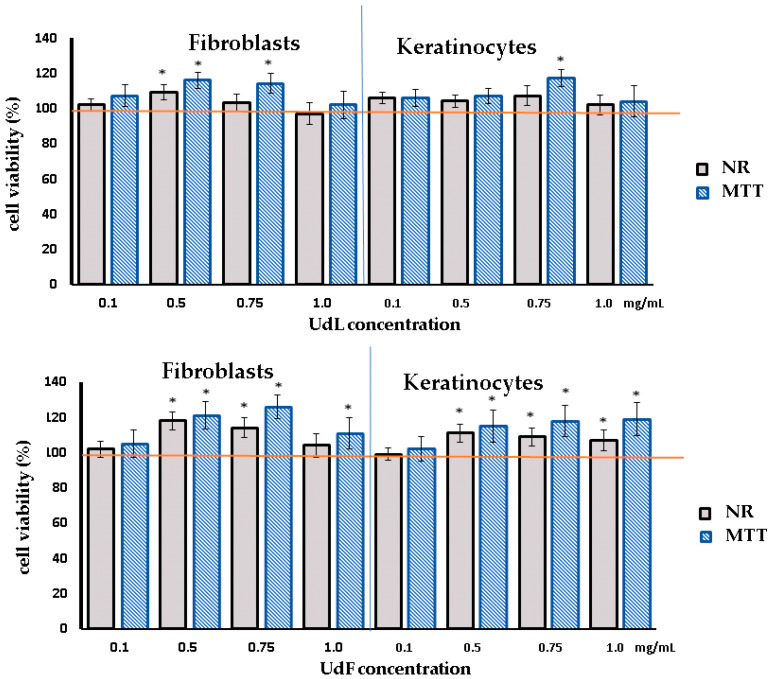
The viability of human skin fibroblasts and keratinocytes assessed by MTT and NR assays after 24 h of exposure with different concentrations of the polyphenolic fraction isolated from leaves (UdLs) and from flowers (UdFs); 0.5% DMSO in a medium was used as the control, taken as a 100% (red line). * Means a statistically significant difference (*p* < 0.05) compared to the control, assessed by a one-way ANOVA followed by Dunnett’s post hoc test.

As shown in Figure 2, after 24 h of incubation, no cytotoxic effects were observed. In fact, at concentrations of 0.5 mg/mL for the UdL and 0.50–1 mg/mL for the UdF, a moderate increase in cell proliferation was detected. However, with prolonged incubation (time 48 h), the highest tested concentration of the UdL led to a slight decrease in the number of viable cells and reduced the cellular oxidoreductase activity (Figure S3).

### 2.3. Antioxidant Tests

#### 2.3.1. Chemical Test

The most common methods for the assessment of antioxidant activity are based on chemical reactions with different radicals or oxidants [29]. In our study, several tests were conducted to preliminarily assess the antioxidant properties of the fraction, including free radical assays based on DPPH• and ABTS•+ radical cations, as well as tests measuring reducing power, such as the FRAP (Ferric Reducing Antioxidant Power) and CUPRAC (Cupric Reducing Antioxidant Capacity). The TPC test, which measures the ability to reduce the Folin–Ciocalteu reagent and is associated with the presence of phenolic hydroxyl groups, was also included. The results summarized in Table 2 show a moderate free radical scavenging potential and reducing power for both fractions. As expected, the UdL was more effective than the UdF because it contained more phenolic compounds.

#### 2.3.2. Intracellular ROS Scavenging Activity

**Figure 3:**
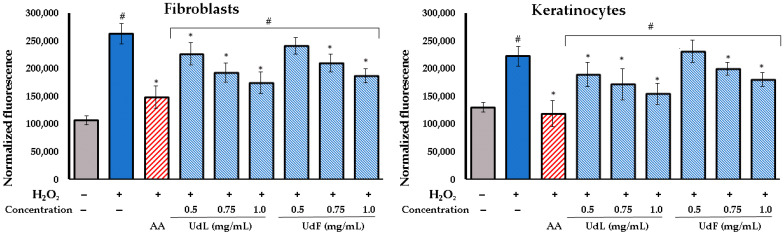
Reactive oxygen species level in H_2_O_2_-treated fibroblast and keratinocytes incubated simultaneously with different concentration of polyphenolic fraction isolated from leaves (UdLs) and from flower (UdF). Control included untreated cells; AA–ascorbic acid, * means a statistically significant difference (*p* < 0.05) compared to H_2_O_2_-treated cells, and # means statistically significant difference (*p* < 0.05) compared to AA as determined by one-way ANOVA followed by Dunnett’s post hoc test.

Further, the ROS scavenging potential of the fractions in skin cells was investigated. The analysis included the measurement of reactive oxygen species based on their reaction with 2′,7′-dichlorodihydrofluorescein diacetate (H_2_DCFDA). This dye is oxidized by ROS, forming a fluorescent compound known as 2′,7′-dichlorofluorescein (DCF). Keratinocytes and fibroblasts were exposed to hydrogen peroxide (H_2_O_2_) to induce oxidative stress. The positive control consisted of cells treated simultaneously with H_2_O_2_ and ascorbic acid, while the negative control included untreated cells, demonstrating the natural ROS levels in the cells. The results obtained, expressed as the normalized fluorescence, are shown in Figure 3.

The results obtained demonstrate that the H_2_O_2_ treatment significantly increased ROS levels in both fibroblasts and keratinocytes. The treatment with ascorbic acid (AA) showed the most pronounced reduction in ROS levels, and none of the tested concentrations reached the ROS level achieved by AA. However, the co-incubation with polyphenolic fractions from leaves (UdLs) and flowers (UdFs) effectively reduced ROS in a concentration-dependent manner. UdLs exhibited a slightly greater antioxidant activity compared to UdFs, which is consistent with the results from chemical tests. These findings suggest that both UdL and UdF fractions possess protective effects against oxidative stress in skin cells.

#### 2.3.3. Prevention Against H_2_O_2_-Induced Cytotoxicity

**Figure 4:**
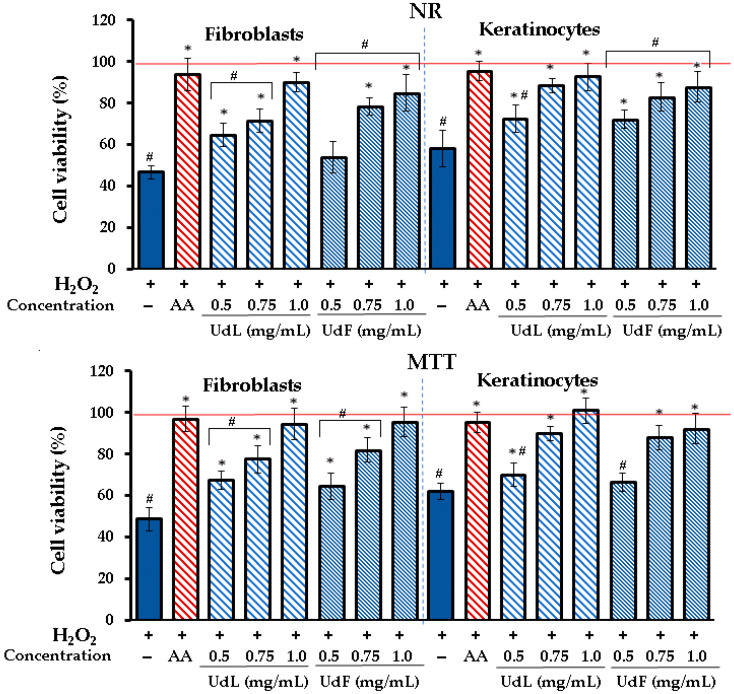
The viability of human skin fibroblasts and keratinocytes treated with H_2_O_2_ and the different concentrations of leaves (UdLs) and flower (UdF) fractions assessed by NR and MTT assays. AA—ascorbic acid (50 µM); * means a statistically significant difference (*p* < 0.05) compared to H_2_O_2_-treated cells; # means a statistically significant difference (*p* < 0.05) compared to AA as determined by the one-way ANOVA followed by Dunnett’s post hoc test; and 0.5% DMSO in a medium was used as a control taken as a 100% (red line).

The protective effects of UdL and UdF extracts on the viability of fibroblasts and keratinocytes subjected to oxidative stress induced by hydrogen peroxide (H_2_O_2_) were assessed using NR and MTT tests. Figure 4 illustrates the obtained results.

In fibroblasts and keratinocytes, the exposure to H_2_O_2_ significantly reduced the cell viability to 47 and 54% in the NR test and to 49% and 65% in the MTT test, respectively. However, the addition of UdL and UdF extracts restored the cell viability in a concentration-dependent manner, with the highest protection observed at 1.0 mg/mL. Only the 0.5 mg/mL concentration of UdFs showed low or no protective activity. Both extracts significantly improved the cell survival compared to the H_2_O_2_-only group. Results from both NR and MTT assays were consistent, although the MTT assay generally showed slightly higher viability percentages.

#### 2.3.4. Impact on Antioxidant Enzymes

In addition to their direct antioxidant activity, which involves reacting with free radicals or the reducing capacity, polyphenols may also influence redox systems within cells. Therefore, in the next step, the effect of the UdL and UdF on superoxide dismutase (SOD) and catalase (CAT) was investigated. Additionally, the impact of the fraction on lipid peroxidation was evaluated by measuring malondialdehyde (MDA) as an indicator of this process.

**Figure 5:**
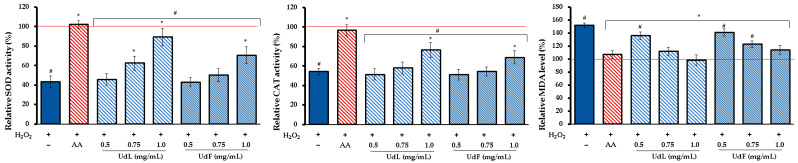
The effect of the simultaneous treatment with H_2_O_2_ and different concentrations of leaf (UdL) and flower (UdF) fractions on antioxidant enzyme activity, expressed as a percentage relative to the untreated control (100%), indicated by the red line; SOD—superoxide dismutase (SOD); CAT—catalase, MDA—malondialdehyde; and AA—ascorbic acid (50 µM). * Indicates a statistically significant difference (*p* < 0.05) versus the H_2_O_2_-treated cells. # means a statistically significant difference (*p* < 0.05) compared to AA.

It has been found (Figure 5) that the exposure to H_2_O_2_ significantly reduced the activity of both SOD and CAT compared to the untreated control, indicating the depletion of these enzymes under oxidative stress conditions. The treatment with ascorbic acid (AA), used as a positive control, restored the enzyme activity to near baseline levels. Both UdL and UdF fractions at the highest concentration increased the activity of SOD and CAT, although the enzyme activity did not reach the levels observed with AA. At lower concentrations, no significant improvement was observed compared to the H_2_O_2_-treated group, with the exception of 0.75 mg/mL of the UdL. Regarding lipid peroxidation, the H_2_O_2_ treatment markedly elevated MDA levels, confirming the enhanced oxidative damage to cellular lipids. The co-treatment with AA significantly reduced MDA levels, bringing them close to control values. Similarly, both UdL and UdF fractions attenuated the H_2_O_2_-induced lipid peroxidation. The observed decreases in MDA levels were statistically significant at all tested concentrations.

### 2.4. Anti-Inflammatory Activity

**Figure 6:**
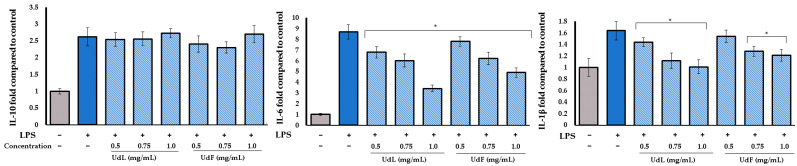
The effect of different concentrations of polyphenolic fractions isolated from leaves (UdLs) and from the flower (UdF) on inrerleukin-10 (IL-10), interleukin-6 (IL-6), and interleukin 1β (IL-1β). The control included untreated cells; * means a statistically significant difference (*p* < 0.05) compared to LPS-treated cells, as determined by the one-way ANOVA followed by Dunnett’s post hoc test.

To evaluate the anti-inflammatory potential, the concentrations of pro-inflammatory interleukins (IL-1β and IL-6) and the anti-inflammatory interleukin (IL-10) were measured in human fibroblast cells stimulated with bacterial lipopolysaccharide (LPS) and H_2_O_2_. The LPS-induced inflammation primarily reflects acute, pathogen-driven immune responses, while the H_2_O_2_-induced inflammation model reflects immune responses associated with oxidative stress. As illustrated in Figure 6, LPS strongly stimulates cytokine production, significantly increasing the expression of all measured cytokines compared to the untreated control. The co-treatment with leaf (UdL) and flower (UdF) polyphenolic fractions of *U. dioica* modulated the inflammatory response in a concentration-dependent manner. Both UdL and UdF fractions significantly reduced the IL-6 expression at all tested concentrations. A similar trend was observed for IL-1β, with all treatments showing significant reductions, except the UdF at 0.5 mg/mL. None of the tested concentrations enhanced or suppressed the IL-10 expression.

**Figure 7:**
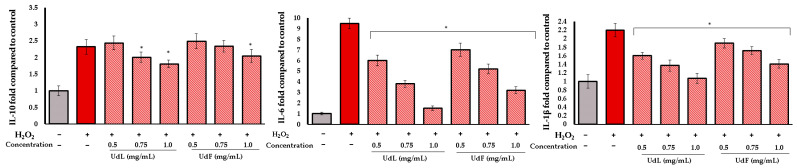
The effect of different concentrations of polyphenolic fractions isolated from leaves (UdLs) and from the flower (UdF) on inrerleukin-10 (IL-10), interleukin-6 (IL-6), and interleukin 1β (IL-1β). The control included untreated cells; * means a statistically significant difference (*p* < 0.05) compared to H_2_O_2_-treated cells, as determined by the one-way ANOVA followed by Dunnett’s post hoc test.

In the case of H_2_O_2_-induced inflammation, the effect was significantly more pronounced. Both the UdL and UdF reduced the levels of all tested cytokines, including IL-10. The highest tested concentration of the UdL decreased IL-6 and IL-1β levels almost to those of the control group. A weaker, but still significant, anti-inflammatory effect was also observed for the UdF (Figure 7).

## 3. Discussion

Many studies have shown that *Urtica dioica* is a valuable medicinal plant characterized by a rich content of various bioactive compounds, particularly from the class of secondary metabolites. In our study, two parts of the plant, leaves and flowers, were used to prepare polyphenol-rich fractions from extracts obtained using accelerated solvent extractions. Consistent with previous reports [30], chlorogenic acid and caffeoylmalic acid were identified as the main constituents of the leaf extract. In addition to chlorogenic acid, the fraction obtained from flowers was also rich in flavonoid compounds, with isorhamnetin derivatives being predominant. The profile of the leaf-derived fraction (UdF) was similar to that established in our previous study [31]; however, the fraction isolated from the extract obtained using the ultrasound-assisted extraction (UAE) contained higher amounts of conjugated derivatives, including caffeoylglucaric acid, caffeoylshikimic acid, p-coumaroylquinic acid, p-coumaroylmalic acid, and feruloylquinic acid. Meanwhile, the fraction obtained from the ASE extract showed higher levels of free phenolic acids, such as caffeic, *p*-coumaric, and ferulic acids. This suggests that the drastic conditions in the ASE promote the breakdown of labile ester bonds. On the other hand, these conditions appear to enhance the extraction efficiency of flavonoid compounds and chlorogenic acid compared to the UAE [31]. The superior performance of the ASE is attributed to the combination of the elevated temperature and pressure, which increases the solubility and mass transfer rates of target compounds. These conditions facilitate the disruption of plant cell walls and the cleavage of ester and glycosidic bonds, resulting in the release of bound phenolic compounds embedded in the cell walls. Furthermore, the ASE reduces the extraction time and solvent consumption, as high yields can be achieved in a single extraction step. The effectiveness of the ASE in extracting polyphenolic compounds has been well documented in the literature for various plant matrices [26,32,33,34].

The presence of polyphenols in the nettle extract contributes to the strong antioxidant effects observed in our study in chemical tests, including FRAP and CUPRAC, and free radical scavenging assays, such as DPPH and ABTS, which is in agreement with the literature data on nettle extracts [35]. Both fractions also demonstrated a potent ROS-scavenging activity in the H_2_DCFDA assay and a significantly reduced ROS production under oxidative stress conditions. They also protect against H_2_O_2_-induced cytotoxicity. These effects were accompanied by an influence on antioxidant enzymes, including superoxide dismutase (SOD) and catalase (CAT). The results indicate that the antioxidant potential of the UdL and UdF is related both to their direct free-radical-neutralizing capacity and to the modulation of the endogenous antioxidant enzyme activity. An in vivo study by Vajic et al. also indicates the ability of nettle to alleviate oxidative stress [30,36]. Moreover, in our previous work, it was demonstrated that the leaf extract reduced ROS levels in human skin fibroblasts under oxidative stress induced by H₂O₂ treatments [23].

Antioxidant activity is especially desirable in the context of skincare. It is well known that excessive levels of reactive oxygen species (ROS) initiate the degradation of the extracellular matrix (ECM) and collagen, resulting in changes to the skin’s structure and function. ROS also contribute to cellular damage, triggering the production of pro-inflammatory cytokines and thereby promoting a chronic low-grade inflammation known as “inflammaging”. All of these factors accelerate skin aging [37,38]. Antioxidants, such as phenolic compounds, may help counteract these effects and potentially slow down the aging process. They can directly react with free radicals and support the endogenous antioxidant enzyme system [39,40]. For example, a dual mechanism of action has been demonstrated for chlorogenic acid, the predominant constituent of the isolated fractions, which not only shows antioxidant properties but also influences SOD, CAT, and GSH-Px [41,42,43]. Liang et al. found that chlorogenic acid is able to activate the Nrf2–Keap1–ARE signaling pathway, a central regulator of the cellular antioxidant response. The activation of Nrf2 leads to the transcription of numerous antioxidant and cytoprotective genes [44]. Isorhamnetin and its glycosides, identified as the main flavonoids in UdFs, also possess strong antioxidant properties and demonstrate ROS-scavenging activity in various cell models with induced oxidative stress [45,46]. In addition, they have been shown to reduce MDA levels and modulate SOD activity, indicating their multidirectional antioxidant effects [47,48]. Furthermore, isorhamnetin has been reported to inhibit oxidative-stress-induced apoptosis by attenuating mitochondrial dysfunction and downregulating pro-apoptotic signaling pathways, including the MAPK and NF-κB pathways [49,50]. Moreover, recent studies suggest that both chlorogenic acid and isorhamnetin may exert anti-inflammatory effects through the suppression of pro-inflammatory cytokines and the inhibition of the iNOS and COX-2 expression, which are often upregulated during oxidative stress. These synergistic actions underscore the therapeutic relevance of these compounds in oxidative stress-related disorders [50].

Furthermore, our research showed that UdL and UdF extracts are non-toxic to human skin fibroblasts and keratinocytes, even after prolonged incubation, which is in agreement with other reports demonstrating the lack of cytotoxicity of *U. dioica* on normal cells [23,51,52]. Moreover, the co-incubation with LPS and the extracts reduced the levels of pro-inflammatory cytokines, including IL-1β and IL-6, induced by LPS, indicating that *U. dioica* fractions can attenuate LPS-induced inflammation. The extracts also effectively suppressed IL-1β, IL-6, and IL-10 in H_2_O_2_-induced inflammation. This model reflects immune responses associated with oxidative stress and may be useful for investigating the anti-aging potential. The anti-inflammatory properties of nettle have also been observed by other researchers, such as Obertreis et al. [53], who found that *U. dioica* extract inhibits cyclooxygenase and lipoxygenase—enzymes responsible for the increased production of pro-inflammatory mediators, such as prostaglandins and leukotrienes. Furthermore, the extract reduces levels of the pro-inflammatory cytokines TNF-α and IL-1 [53]. In addition, clinical studies have demonstrated that *U. dioica* may be beneficial in the treatment of inflammation-related diseases [54,55].

Similarly to its antioxidant and cytoprotective properties, the anti-inflammatory activity of the UdL and UdF may be attributed to their abundance of phenolic acids and flavonoids [56,57]. Chlorogenic acid, for instance, has been reported to suppress the production of several pro-inflammatory cytokines, including IL-1β, IL-6, TNF-α, and IL-8, which is mediated by nuclear factor (NF)-κB and p38 signaling pathways [41,44,58]. In addition to cytokine suppression, chlorogenic acid has been shown to inhibit the activation of inflammasomes such as NLRP3, further reducing the release of mature IL-1β and contributing to its anti-inflammatory efficacy. It also attenuates leukocyte infiltration and edema formation in in vivo models of inflammation [59]. In turn, caffeic acid has been shown to influence the expression of cyclooxygenase, prostaglandins, and interleukins [60,61,62]. Many flavonoids are also capable of reducing inflammation and the anti-inflammatory activity is associated with the modulation of various signaling pathways. It has been shown that isorhamnetin derivatives can inhibit key proteins in the mitogen-activated protein kinase (MAPK) pathways, including JNK and p38, in LPS-induced RAW264.7 macrophage cells [63]. Moreover, these compounds may suppress nitric oxide (NO) production, a molecule known to contribute to inflammation and tissue damage [64]. The anti-inflammatory effects are also mediated through the inhibition of the nuclear factor kappa B (NF-κB) pathway [65], a central regulator of inflammatory responses. Furthermore, several studies have demonstrated that isorhamnetin derivatives inhibit the production of pro-inflammatory cytokines and mediators, including TNF-α, IL-1β, and IL-6, supporting their potential role as modulators of immune and inflammatory processes [64,66,67,68].

In general, polyphenolic compounds are also known to modulate the Nrf2/Keap1 signaling pathway, which plays a central role in the cellular defense against oxidative stress [69]. Upon activation, Nrf2 translocates to the nucleus and induces the expression of antioxidant and cytoprotective genes, including HO-1, NQO1, and various glutathione-related enzymes. By disrupting the interaction between Nrf2 and its inhibitor Keap1, polyphenols can promote the sustained activation of this pathway, thereby enhancing the cellular resilience to oxidative insults. Moreover, the activation of Nrf2 has been linked not only to antioxidant responses but also to anti-inflammatory effects, as it can suppress pro-inflammatory cytokine production and inhibit NF-κB signaling [69]. This may further explain the antioxidant and anti-inflammatory properties of the polyphenolic fraction from *U. dioica*.

Taking into account the results of our study and the existing literature, it can be concluded that the significant antioxidant and anti-inflammatory activity of *U. dioica* is related to the presence of polyphenolic compounds.

## 4. Materials and Methods

### 4.1. Extract Preparation and Phytochemical Characterization

*Urtica dioica* plants collected in the Botanical Garden in Lublin were washed, separated into leaves and flowers, and then freeze-dried (Christ Alpha 2–4 LDplus dryer, Martin Christ Gefriertrocknungsanlagen, GmbH, Osterode am Harz, Germany). Plant materials were pulverized and subjected to accelerated solvent extraction (Dionex—ASE—350 Accelerated Solvent Extractor, Thermo Fisher Scientific Inc., Sunnyvale, CA, USA) using 80% ethanol. The extraction conditions were based on the study by Repajić et al. [26] and were as follows: an extraction temperature of 110 °C, a static extraction time of 10 min, and three extraction cycles. Polyphenolic fraction from extract was isolated using solid-phase extraction in LiChrolut^®^ RP-18 SPE tubes (Merck, Darmstadt, Germany).

Separation of the extracts was performed using Titan column (10 cm length × 2.1 mm i.d., 1.9 µm particle size) (Supelco, Sigma-Aldrich, Burlington, MA, USA). The mobile-phase composition, gradient elution program, and MS parameters were applied as previously described [70]. Detection and identification of components were based on MS data acquired in negative ionization mode and registered UV spectra. All standards and reagents were obtained from Sigma-Aldrich (St. Louis, MO, USA). Detailed information on the quantitative analysis is provided in Table S1.

### 4.2. Antioxidant Tests

#### 4.2.1. Free Radical Scavenging Activity

For the ABTS assay, a mixture of 7 mM ABTS solution and 2.4 mM potassium persulfate was prepared in a 1:1 ratio and appropriately diluted. Then, 10 μL of the extract at various concentrations was mixed with 190 μL of the ABTS/potassium persulfate solution in 96-well plate. The plate was incubated in the dark for 30 min, and the absorbance was measured at 734 nm using a UV/VIS spectrophotometer (Thermo Fisher Scientific, Waltham, MA, USA). In the DPPH (1,1-diphenyl-2-picrylhydrazyl) assay, extracts at various concentrations and 100 μL of a 0.4 mM DPPH methanol solution were mixed in 96-well plate for 15 min. The plate was placed in a plate reader, and absorbance was measured at λ = 517 nm. Trolox and ascorbic acid were used as positive controls.

#### 4.2.2. Total Polyphenols Content (TPC)

In each well, 25 μL of the extract, 25 μL of a ninefold diluted Folin–Ciocalteu (FC) reagent, and 200 μL of distilled water were combined. After a 5 min incubation period, 25 μL of saturated sodium carbonate (Na_2_CO_3_) solution was added. The prepared plate was then incubated in the dark at room temperature for 60 min. Absorbance of the samples was recorded at a wavelength of 750 nm. The results were reported as milligrams of gallic acid equivalents per gram of sample (mg GAE/g).

#### 4.2.3. Ferric Ion Reducing Antioxidant Power Assay

A total of 180 μL of FRAP solution (0.3 M acetate buffer, 0.01 M tripyridyltriazine 0.02 M FeCl_3_ × 6H_2_O in a 10:1:1 *v*/*v* ratio) was mixed with different concentrations of the extract. The mixture was incubated for 20 min, and absorbance was measured at 593 nm using a plate reader.

#### 4.2.4. Cupric Ion Reducing Antioxidant Capacity Assay

The CUPRAC assay was conducted by mixing the sample extracts with 190 μL of a working reagent composed of acetate buffer (pH 7.0), neocuproine solution (7.5 mM), and CuCl_2_ solution (10 mM) in equal volumetric proportions (1:1:1). The resulting mixture was incubated for 15 min at room temperature, after which the absorbance was measured at 450 nm. The antioxidant capacity was expressed as milligrams of Trolox equivalents per gram of sample (mg Trolox/g sample).

### 4.3. Biological Assays

#### 4.3.1. Cell Cultures

Fibroblasts (HDF) and keratinocytes (HaCaT) both obtained from CLS Cell Lines Service (Eppelheim, Germany) were cultured as described previously [71]. The cells were washed twice with Phosphate-Buffered Saline (Biological Industries, Kibbutz Beit-Haemek, Israel). The cells were then trypsinized and placed in fresh DMEM. The cells were seeded in 96-well plates at a density of 1 × 10^4^ cells/well and incubated for 24 h to prepare the cells for cytotoxicity assays.

#### 4.3.2. Cell Viability Assays

MTT Assay: Cells were seeded in 96-well plates and incubated for 24 h with 100 µL of culture medium containing various concentrations of the tested extracts. After incubation, the medium was replaced with fresh medium containing MTT solution (5 mg/mL; 25 µL per well), and cells were incubated for an additional 3 h. Formed formazan crystals were solubilized overnight using a solution of 10% SDS in 0.01 M HCl. Absorbance was measured at 570 nm using a spectrophotometer.

Neutral Red (NR) Uptake Assay: Cells were cultured in 96-well plates for 24 h in 100 µL of medium with the tested extract concentrations. The medium was then replaced with 0.4% NR solution, and the cells were incubated for 3 h at 37 °C in a humidified 5% CO_2_ atmosphere. After removing the dye, cells were fixed with 1% CaCl_2_ in 4% paraformaldehyde (200 µL). The absorbed dye was extracted using 1% acetic acid in 50% ethanol (100 µL). Plates were gently shaken for 20 min at room temperature, and absorbance was recorded at 540 nm.

#### 4.3.3. Intracellular ROS Scavenging Activity

The intracellular levels of reactive oxygen species (ROS) in human fibroblasts and keratinocytes were measured using the fluorogenic probe H_2_DCFDA. To induce oxidative stress, hydrogen peroxide (H_2_O_2_) was added to each well at a final concentration of 500 µM. Subsequently, the cells were cultured in the presence of various concentrations of the tested extracts. ROS production in the treated skin cells was quantified spectrofluorometrically by measuring fluorescence at an excitation wavelength of 485 nm and an emission wavelength of 530 nm, following 60 min of incubation with the probe.

#### 4.3.4. SOD, CAT, and MDA Levels.

Cells were exposed to oxidative stress by treatment with hydrogen peroxide (H_2_O_2_) at a final concentration of 500 μM. Simultaneously, they were incubated with the test extracts. Following 6 h of incubation at 37 °C under standard culture conditions (5% CO_2_, humidified atmosphere), the levels of malondialdehyde (MDA), as well as the enzymatic activities of superoxide dismutase (SOD) and catalase (CAT), were evaluated. Quantification was carried out using commercially available assay kits (Abcam, Berlin, Germany), according to the manufacturers’ instructions.

#### 4.3.5. Anti-Inflammatory Tests

Anti-inflammatory effects were assessed by measuring IL-1β, IL-6, and IL-10 using ELISA kits (Elabscience Biotechnology Inc., Houston, TX, USA). The procedure was performed according to the kit instructions. Fibroblast cells were exposed to bacterial lipopolysaccharide (from Escherichia coli O111:B4) at a concentration of 10 µg/mL [72] or H_2_O_2_ at a concentration of 500 µM simultaneously with the tested samples, for 24 h. Absorbance at 450 nm was then measured using a FilterMax F5 (Thermo Fisher Scientific, Waltham, MA, USA).

## 5. Conclusions

The present study demonstrates that polyphenol-rich fractions obtained from the leaves (UdLs) and flowers (UdFs) of *U. dioica* possess significant antioxidant and anti-inflammatory properties in human skin cell models. Both extracts were well tolerated by human fibroblasts and keratinocytes, showing no cytotoxicity at the tested concentrations. Furthermore, both the UdL and UdF significantly reduced intracellular ROS levels, improved the activity of key antioxidant enzymes (SOD and CAT), and attenuated lipid peroxidation, indicating their strong cytoprotective and redox-modulating potential. Importantly, both fractions were also able to mitigate inflammatory responses in fibroblasts stimulated with bacterial lipopolysaccharide (LPS), significantly decreasing the levels of pro-inflammatory cytokines IL-1β and IL-6 without affecting the expression of the anti-inflammatory cytokine IL-10. Furthermore, they suppressed IL-1β, IL-6, and IL-10 in oxidative stress-related inflammation.

Taken together, these findings highlight the potential of *U. dioica* extracts as multifunctional agents that can protect skin cells from oxidative and inflammatory damage. Their favorable safety profile, natural origin, and proven biological activity make them attractive candidates for use in cosmeceuticals and dermatological formulations aimed at supporting skin health, delaying the aging process, and aiding in the management of inflammatory skin disorders. Future research is warranted to further explore their therapeutic potential and optimize formulation strategies for an effective topical delivery.

## Figures and Tables

**Table 1:** Mass spectrometric data of the predominant constituents and the results of the quantitative analysis (mg/g ± standard deviation) of the fractions isolated from the *Urtica dioica* flower (UdF) and leaves (UdLs).

RT (min)	Mass Data (*m*/*z*–H)	Fragment (*m*/*z*–H)	Formula	Δ ppm	Component	UdL	UdF
5.60	315.07261	(152)	C_13_H_16_O_9_	1.44	Dihydroxybenzoic acid hexoside	0.21 ± 0.01	nd
6.22	371.06188	(209,135,179)	C_15_H_16_O_11_	−0.28	Caffeoylglucaric acid	2.21 ± 0.08	nd
7.31	315.07289	(152)	C_13_H_16_O_9_	2.32	Dihydroxybenzoic acid hexoside	0.18 ± 0.01	nd
13.82	353.08887	(191,135,179)	C_16_H_18_O_9_	3.01	5-*O*-caffeoylquinic acid *	35.12 ± 1.11	17.51 ± 0.98
15.08	179.03551	(135)	C_9_H_8_O_4_	2.93	Caffeic acid *	4.09 ± 0.20	0.32 ± 0.01
15.68	353.08863	(191,135,179)	C_16_H_18_O_9_	2.33	4-*O*-caffeoylquinic acid *	0.37 ± 0.03	0.11 ± 0.01
16.80	295.04614	(133,135,179)	C_13_H_12_O_8_	0.67	Caffeoylmalic acid	27.18 ± 1.53	2.49 ± 0.11
20.43	163.04071		C_9_H_8_O_3_	3.92	*p*-coumaric acid *	4.21 ± 0.22	nd
23.65	193.05011		C_10_H_10_O_4_	−2.67	Ferulic acid *	1.52 ± 0.05	nd
28.47	609.14562	(300,463)	C_27_H_30_O_16_	−0.80	Quercetin-3-*O*-rutinoside *	1.51 ± 0.04	1.94 ± 0.95
29.22	463.08785	(300)	C_21_H_20_O_12_	-0.75	Quercetin 3-*O*-glucoside *	0.21 ± 0.01	1.11 ± 0.18
32.13	505.10076	(463,300)	C_23_H_22_O_13_	3.94	Quercetin acetylglucoside	0.18 ± 0.00	0.96 ± 0.04
34.55	593.14999	(285)	C_27_H_30_O_15_	−2.03	Kaempferol 3-*O*-rutinoside *	0.09 ± 0.00	0.24 ± 0.01
36.05	623.16183		C_28_H_32_O_16_	0.11	Isorhamnetin 3-*O*-rutinoside *	0.01 ± 0.00	0.10 ± 0.00
36.64	477.10394	(315)	C_22_H_22_O_12_	0.19	Isorhamnetin-3-*O*-glucoside	0.05 ± 0.01	1.94 ± 0.17
38.91	489.10519	(284)	C_23_H_22_O_12_	2.73	Kaempferol acetylglucoside	nd	0.29 ± 0.01
40.92	519.11690	(315)	C_24_H_24_O_13_	4.78	Isorhamnetin acetylglucoside	nd	4.93 ± 0.25

* Identification was confirmed using standard; nd—not detected.

**Table 2:** Antioxidant activity of *Urtica dioica* polyphenolic fractions isolated from leaves (UdLs) and flower (UdF).

	DPPH *	ABTS	FRAP	CUPRAC	TPC
UdL	78.56 ± 0.85 ^b^	8.37 ± 0.28 ^b^	15.53 ± 0.82 ^b^	59.67 ± 1.99 ^b^	37.58 ± 1.92 ^b^
UdF	124.77 ± 1.38 ^c^	20.98 ± 0.12 ^c^	10.70 ± 0.87 ^a^	38.22 ± 1.28 ^a^	24.08 ± 1.02 ^a^
AA	25.55 ± 0.20 ^a^	4.24 ± 0.23 ^a^			

DPPH and ABTS values show IC50 (µg/mL); FRAP, and CUPRAC values are shown in percentages; TPC values are expressed as mgGAE/g; and different letters indicate a statistically significant difference.

## Data Availability

The data presented in this study are available on request from the corresponding author.

## Supplementary Materials

The following supporting information can be downloaded at https://www.mdpi.com/article/10.3390/molecules30122515/s1: Table S1: Detailed information on the quantitative analysis. Figure S1: Chromatogram obtained at 350 nm of the polyphenolic fraction isolated from ethanol-water extract of *U. dioica* leaves (green line) and flowers (red line). Figure S2: MS and UV spectra of the main components identified in *Urtica dioica*. Figure S3: The viability of human skin fibroblasts and keratinocytes assessed by MTT and NR assays after 48 h of exposure with different concentration of polyphenolic fraction isolated from leaves (UdL) and from flowers (UdFs).
