# Supplementary material for: Oxidative Stress Protection and Anti-Inflammatory Activity of Polyphenolic Fraction from Urtica dioica: In Vitro Study Using Human Skin Cells
Source: Molecules. 2025 Jun 9;30(12):2515. doi: 10.3390/molecules30122515 (PMC12195803; doi:10.3390/molecules30122515)
Supplement: Supplementary file 1 [file molecules-30-02515-s001.zip › molecules-3658607-supplementary.pdf]

Oxidative Stress Protection and Anti-Inflammatory Activity of Polyphenolic Fraction from *Urtica dioica*: In Vitro Study Using Human Skin Cells

Katarzyna Wójcik-Borowska [1], Weronika Wójciak [2], Magdalena Żuk [2], Piotr Luchowski [3], Agnieszka Skalska-Kamińska [2], Wiktoria Pacuła [2], Ireneusz Sowa [2] and Magdalena Wójciak [2,*]

[1] Department of Child Neurology, Medical University of Lublin, 20-093 Lublin, Poland; kwojcikborowska@gmail.com
[2] Department of Analytical Chemistry, Medical University of Lublin, Chodźki 4a, 20-093 Lublin, Poland; weronikawojciak01@gmail.com (W.W.); magdalena.zu25@gmail.com (M.Ż.); agnieszka.skalska-kaminska@umlub.pl (A.S.-K.); wiktoria.pacula@umlub.pl (W.P.); i.sowa@umlub.pl (I.S.)
[3] Department of Neurology and Neurological Nursing, Medical University of Lublin, 20-954 Lublin, Poland; piotr.luchowski@umlub.pl
* Correspondence: magdalena.wojciak@umlub.pl

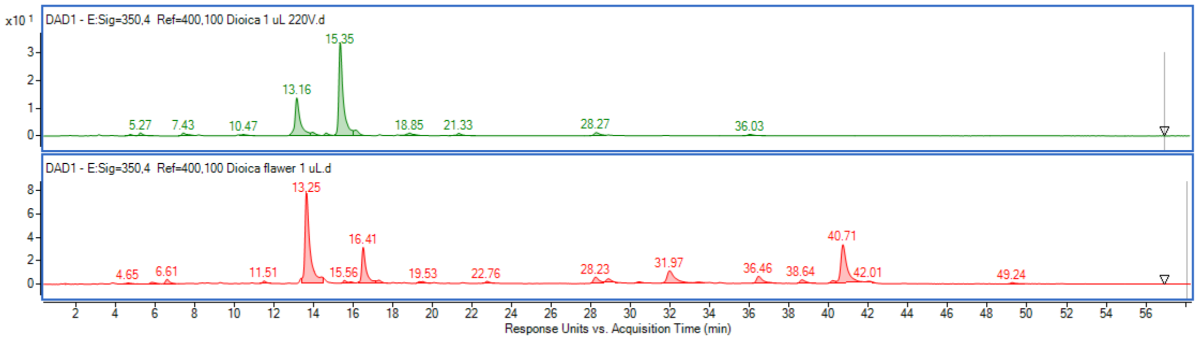

Figure S1. Chromatogram obtained at 350 nm of the polyphenolic fraction isolated from ethanol-water extract of *U. dioica* leaves (green line) and flowers (red line).

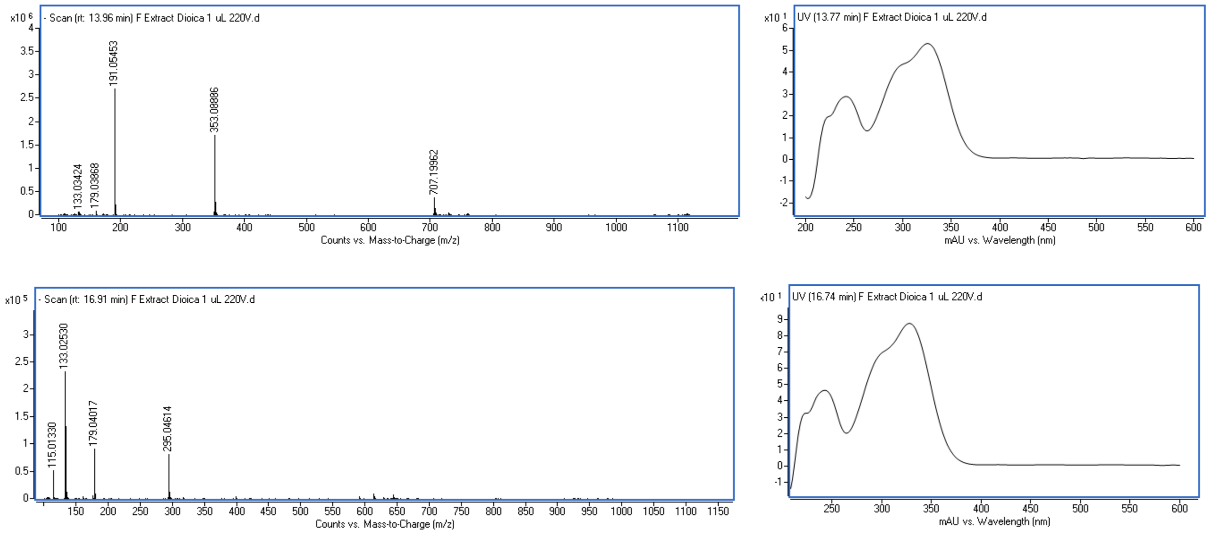

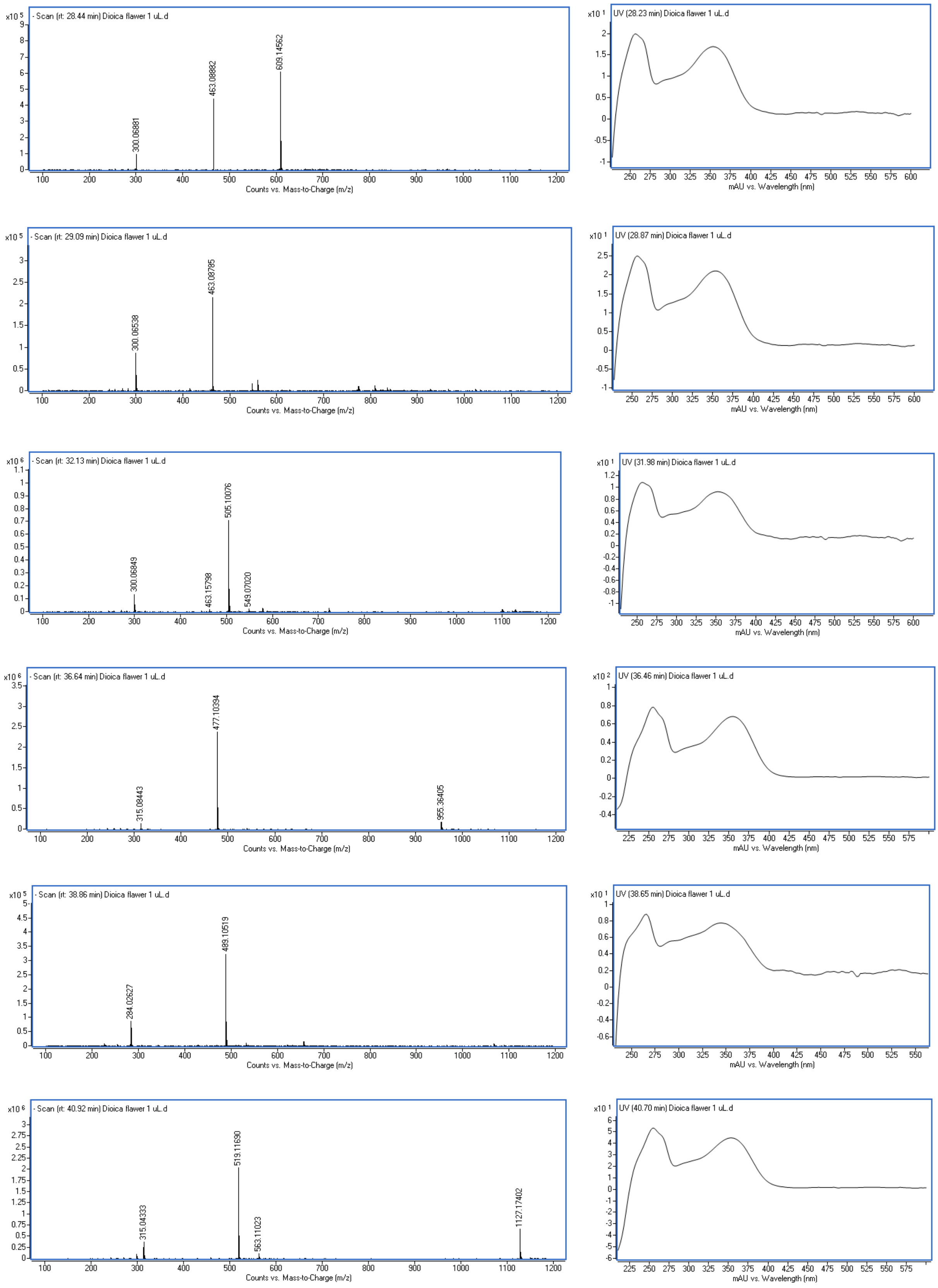

Figure S2. MS and UV spectra of the main components identified in *Urtica dioica*.

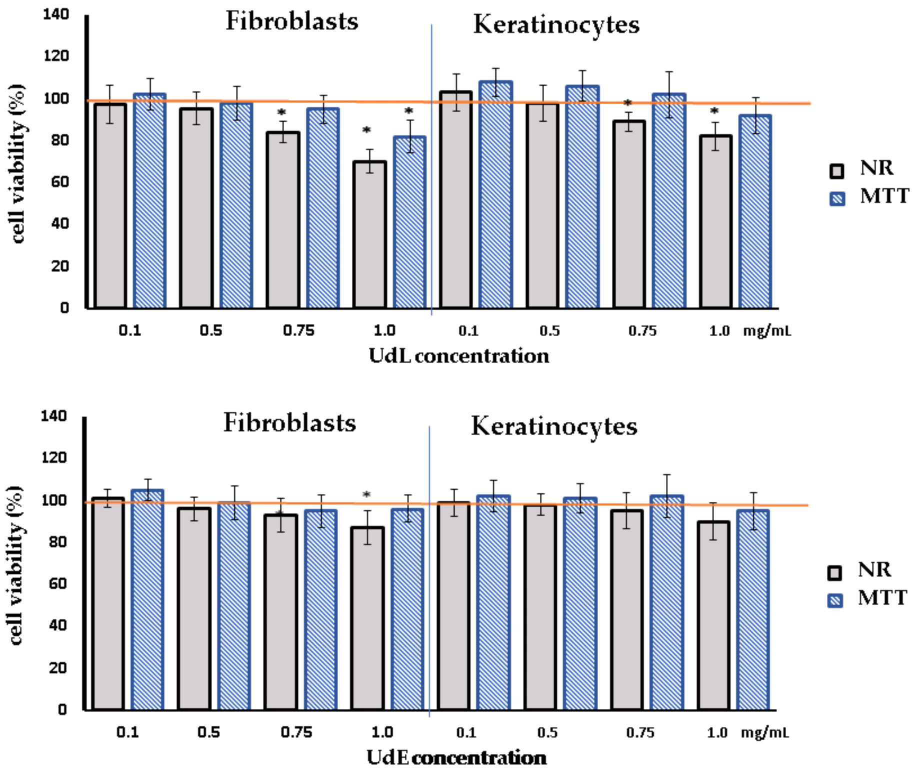

Figure S3. The viability of human skin fibroblasts and keratinocytes assessed by MTT and NR assays after 48 h of exposure with different concentration of polyphenolic fraction isolated from leaves (UdL) and from flower (UdF). 0.5% DMSO in medium was used as control taken as a 100% (red line). * means a statistically significant difference ($p < 0.05$) assessed by one-way ANOVA followed by Dunnett's post hoc test.

Table S1. Detailed information on the quantitative analysis

Compound	Wavelength	Calibration range	Calibration curve
Dihydroxybenzoic acid	λ=310 nm	0.003 – 0.03 mg/mL	y = 9955x + 6.3
5-O-caffeoylquinic acid*	λ=325 nm	0.064 – 0.64 mg/mL	y = 8871.8x – 3.3
4-O-caffeoylquinic acid	λ=325 nm	0.005 – 0.05 mg/mL	y = 8665x – 1.03
Caffeic acid	λ=325 nm	0.005 – 0.05 mg/mL	y= = 17222x + 25.1
p-coumaric acid	λ=310 nm	0.006 – 0.06 mg/mL	y= 31515x – 12.6
Ferulic acid	λ=325 nm	0.002 – 0.02 mg/mL	y = 15583x – 2.4
Quercetin-3-*O*-rutinoside	λ=350 nm	0.003 – 0.03 mg/mL	y = 6147.1x – 2.2
Quercetin 3-*O*-glucoside	λ=350 nm	0.002 – 0.02 mg/mL	y = 16546x – 2.1
Kaempferol 3-*O*-rutinoside	λ=350 nm	0.0001–0.001 mg/mL	y = 5609.5x – 0.17
Isorhamnetin 3-*O*-rutinoside	λ=350 nm	0.006 – 0.06 mg/mL	y = 2397.2x + 5.4
Isorhamnetin 3-*O*-rutinoside	λ=350 nm	0.0001–0.001 mg/mL	y = 2371.7x – 0.15

dihydroxybenzoic acid hexosides were calculated based on calibration curve for 2,5-dihydroxybenzoic acid; caffeoylglucaric acid and caffeoylmalic acid were calculated based on calibration curve for caffeic acid; acetylglucosides were calculated based on calibration curve for corresponding glucosides; Isorhamnetin-3-O-glucoside was calculated based on calibration curve for isorhamnetin 3-O-rutinoside; * - for quantification the sample was diluted 1:10 v/v.
